# The molecular view of mechanical stress of brain cells, local translation, and neurodegenerative diseases

**DOI:** 10.18699/VJ21.011

**Published:** 2021-02

**Authors:** T.M. Khlebodarova

**Affiliations:** Institute of Cytology and Genetics of Siberian Branch of the Russian Academy of Sciences, Novosibirsk, Russia Kurchatov Genomic Center of the Institute of Cytology and Genetics of Siberian Branch of the Russian Academy of Sciences, Novosibirsk, Russia

**Keywords:** synapse, YAP/TAZ mechanosensor, mTOR, FMRP-dependent translation, complex dynamics, F actin, WAVE regulatory complex, autism spectrum disorders, epileptic encephalopathy, синапс, механосенсор YAP/TAZ, mTOR, FMRP-зависимая трансляция, сложная динамика, F-актин, WAVE регуляторный комплекс, расстройства аутистического спектра, эпилептическая энцефалопатия

## Abstract

The assumption that chronic mechanical stress in brain cells stemming from intracranial hypertension, arterial hypertension, or mechanical injury is a risk factor for neurodegenerative diseases was put forward in the 1990s and has since been supported. However, the molecular mechanisms that underlie the way from cell exposure to mechanical stress to disturbances in synaptic plasticity followed by changes in behavior, cognition, and memory are still poorly understood. Here we review (1) the current knowledge of molecular mechanisms regulating local translation and the actin cytoskeleton state at an activated synapse, where they play a key role in the formation of various sorts of synaptic plasticity and long-term memory, and (2) possible pathways of mechanical stress intervention. The roles of the mTOR (mammalian target of rapamycin) signaling pathway; the RNA-binding FMRP protein; the CYFIP1 protein, interacting with FMRP; the family of small GTPases; and the WAVE regulatory complex in the regulation of translation initiation and actin cytoskeleton rearrangements in dendritic spines of the activated synapse are discussed. Evidence is provided that chronic mechanical stress may result in aberrant activation of mTOR signaling and the WAVE regulatory complex via the YAP/TAZ system, the key sensor of mechanical signals, and influence the associated pathways regulating the formation of F actin filaments and the dendritic spine structure. These consequences may be a risk factor for various neurological conditions, including autistic spectrum disorders and epileptic encephalopathy. In further consideration of the role of the local translation system in the development of neuropsychic and neurodegenerative diseases, an original hypothesis was put forward that one of the possible causes of synaptopathies is impaired proteome stability associated with mTOR hyperactivity and formation of complex dynamic modes of de novo protein synthesis in response to synapse-stimulating factors, including chronic mechanical stress.

## Abstract (ru)

Идея о том, что хронический механический стресс, который испытывают клетки мозга при повышенном внутричерепном давлении, артериальной гипертензии или вследствие травмы, может быть одним из факторов риска в развитии нейродегенеративных заболеваний, появилась еще в 90-е годы прошлого столетия и поддерживается в настоящее время. Однако молекулярно-генетические механизмы реализации событий, ведущих от механического воздействия на клетки к нарушению пластичности синапсов и последующему изменению поведения, когнитивных способностей и памяти, не ясны. В настоящем обзоре рассмотрены существующие данные о молекулярно-генетических механизмах регуляции локальной трансляции и актинового цитоскелета в активированном синапсе, играющих центральную роль в формировании различных видов пластичности синапса и долговременной памяти, и возможных путях влияния механического стресса на их состояние. Обсуждается роль mTOR сигнального каскада, РНК-связывающего белка FMRP, белка CYFIP1, взаимодействующего с FMRP, семейства малых ГТФаз и WAVE регуляторного комплекса в регуляции инициации локальной трансляции и перестроек актинового цитоскелета в дендритных шипиках активированного синапса. Приводятся факты, свидетельствующие о том, что в условиях хронического механического стресса возможна аберрантная активация mTOR сигнального каскада и WAVE регуляторного комплекса через сенсор механических сигналов – регуляторный фактор YAP/TAZ, следствием которой могут быть нарушения активности системы локальной трансляции, а также связанных с ними механизмов регуляции формирования F-актиновых филаментов и структуры дендритных шипиков. Это может быть одной из причин развития различных неврологических патологий, включая аутистические расстройства и эпилептическую энцефалопатию. Высказывается оригинальная гипотеза, согласно которой одной из возможных причин синаптопатий может быть нарушение стабильности протеома, связанное с гиперактивностью mTOR и формированием сложных динамических режимов синтеза белков de novo в ответ на стимуляцию синапса, в том числе и в условиях хронического механического стресса.

## Mechanical stress and neurodegenerative disorders

Mechanical signals are an important factor that determines the fate of cells, including their proliferation, survival, and differentiation, and takes part in tissue regeneration and wound healing. Mechanotransduction involves the reception of these forces and their conversion to biochemical and molecular signals, in particular, triggering of signaling pathways and expression of certain genes to allow cell adaptation to physical environment. There is ample evidence for the central role of the transcription regulator YAP (yes-associated protein 1) and its paralog TAZ (transcriptional co-activator with PDZbinding motif), collectively named YAP/TAZ, as mechanical signal sensors and mediators (Dupont et al., 2011; Totaro et al., 2018; Dasgupta, McCollum, 2019). Impaired interaction of a cell and its environment causes aberrant YAP/TAZ activation and eventually a variety of diseases: atherosclerosis, fibrosis, lung hypertension, inflammation, muscle dystrophy, and cancer (Levy Nogueira et al., 2015, 2018; Yu et al., 2015; Panciera et al., 2017; Hong et al., 2019; Zhu et al., 2020). Recent studies indicate that mechanical stress may be among the causes of neurodegenerative processes in the brain, e.g., Alzheimer’s disease (Levy Nogueira et al., 2015, 2016a, b, 2018).

The assumption that chronic mechanical stress experienced by brain cells exposed to intracranial hypertension, arterial hypertension, or mechanical injury is a risk factor for Alzheimer’s disease and other neurodegenerative conditions was put forward as early as (Wostyn, 1994), and it is supported still now (Levy Nogueira et al., 2018).

**Fig. 1.**
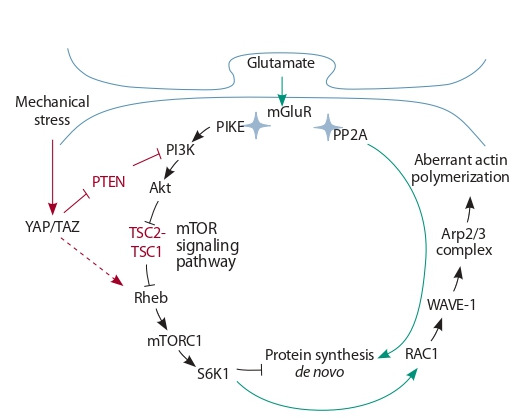
Possible pathways of the effect of mechanical stress mediated by mTOR signaling on the intensity of local translation and the formation of actin cytoskeleton in dendritic spines of glutamatergic synapses in pyramidal cells of the hippocampus mGluR – receptor protein; PIKE (PI3-kinase enhancer), Rheb (Ras homologue enriched in brain), and Rac1 – GTPases; PI3K – phosphatidylinositol-3-kinase; Akt – protein kinase B; TSC1/2 – tuberous sclerosis complex 1/2; mTOR (mechanistic target of rapamycin) – serine/threonine kinase; S6K1 – S6 kinase 1; PTEN – phosphatase and tensin homolog; PP2A – protein phosphatase 2A; YAP/TAZ – mechanosensor; WAVE-1 – WAVE-1 regulatory complex; Arp2/3 – actin binding proteins. Proteins whose gene mutations are associated with neurological disorders are shown in red. Green arrows indicate translation activation via PP2A phosphatase and actin polymerization via S6 kinase and Rac1 GTPase in response to synapse stimulation by glutamate. Red arrows indicate possible mechanisms by which mechanical stress affects mTOR signaling.

What facts point to the existence of mechanisms by which mechanical stress influences nerve cell functions? First, it has been found that YAP/TAZ, being the key sensor and mediator of mechanical signals, activates the mTOR (mammalian target of rapamycin) signaling pathway (Tumaneng et al., 2012; McCarthy, 2013; Hu et al., 2017). This pathway is the central regulator of cap-dependent translation at a synapse. This regulation supports the dynamic plasticity of the synapse in response to external stimuli, and this plasticity underlies learning and memory (Costa-Mattioli et al., 2009; Buffington et al., 2014; Rosenberg et al., 2014; Santini et al., 2014). Disruption of these processes causes synaptic dysfunction and various neuropsychic disorders (Trifonova et al., 2017). YAP/TAZ activates mTOR by two mechanisms, illustrated in Fig. 1: by stimulating the transcription of Rheb GTPase (Ras homologue enriched in brain) (Hu et al., 2017), which is an mTORС1 kinase activator, and by inhibiting the translation of PTEN (phosphatase and tensin homolog) with miR29 microRNA, thereby inducing aberrant PI3K-mediated activation of mTORC1 and mTORC2 kinases (Tumaneng et al., 2012; McCarthy, 2013).

Second, the actin cytoskeleton is the key mediator of mechanical signals (Seo, Kim, 2018). Its rearrangements in dendritic spines contribute much to learning and long-term memory formation (Basu, Lamprecht, 2018; Borovac et al., 2018). They are controlled by Rho GTPases (Tapon, Hall, 1997), whose hypo- or hyperactivity results in dendritic spine structure distortion, defective memory, and poor learnability. It may also cause multiple neurodevelopment disorders of various origins (Ba et al., 2013; Pyronneau et al., 2017; Zamboni et al., 2018; Nishiyama, 2019). The function of Rho GTPases at an activated synapse depends considerably on their de novo synthesis, which is determined by mTOR activity (Briz et al., 2015).

The activation of the mTOR signaling pathway by the YAP/ TAZ mechanosensor under mechanical stress (Tumaneng et al., 2012; McCarthy, 2013; Hu et al., 2017) also promotes the activation of the heteropentameric WAVE regulatory complex (WASP family verprolin homologue) via S6K kinase and RAC1 GTPase (Derivery et al., 2009) by inducing its breakdown into subcomplexes and interaction of WAVE1 with Arp2/3 (Cory, Ridley, 2002; Millard et al., 2004; Abekhoukh, Bardoni, 2014; Molinie, Gautreau, 2018). These processes result in aberrant actin polymerization and structural anomalies of dendritic spines (see Fig. 1). 

Thus, the pathways of the influence of mechanical stimuli on nerve cell functioning may involve the activation of mTOR signaling and rearrangements of the actin cytoskeleton in dendritic spines, which, in turn, depend on the activity of the local translation system at the synapse, controlled by mTOR. Just the disturbances in the local translation system at the synapse, including those caused by enhanced mTOR activity (manifesting themselves as synapse plasticity aberrations in the form of imbalance between synapse excitation and inhibition (Gobert et al., 2020)) are thought to be associated with various neuropsychic conditions, including autism spectrum disorders (ASDs), epilepsy, Parkinson’s disease, and Alzheimer’s disease (Gkogkas, Sonenberg, 2013; Meng et al., 2013; Won et al., 2013; Cai et al., 2015; Huber et al., 2015; Pramparo et al., 2015; Klein et al., 2016; Martin, 2016; Onore et al., 2017). 

In this regard, the molecular mechanisms regulating local translation and dynamic rearrangements of the actin cytoskeleton in dendritic spines affected by this regulation enjoy close attention (Bramham, 2008). 

## Local translation and neurodegenerative disorders

There is convincing evidence that local cap-dependent translation in the postsynaptic space of a dendritic spine enables its dynamic plasticity in response to external stimuli, which underlies learning and memory (Huber et al., 2000; CostaMattioli et al., 2009; Rosenberg et al., 2014; Santini et al., 2014; Louros, Osterweil, 2016). 

**Fig. 2.**
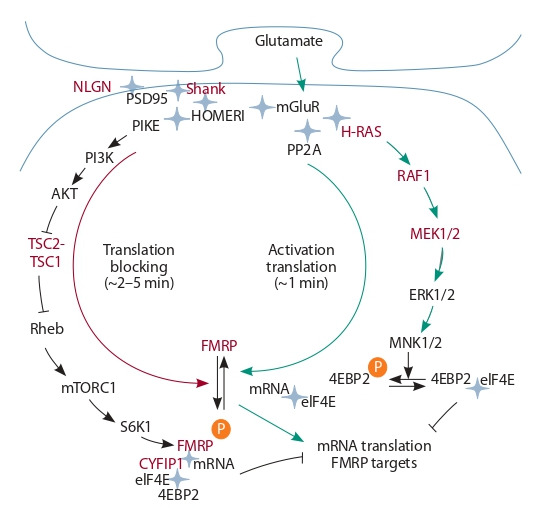
Schematic presentation of local translation regulation in dendritic spines of glutamatergic synapses of hippocampal pyramidal cells in response to synapse stimulation. mGluR – receptor protein; NLGNs, Shank, PSD95, HOMERI – postsynaptic proteins; PIKE (PI3-kinase enhancer) and Rheb (Ras homologue enriched in brain) – GTPases; PI3K – phosphatidylinositol-3-kinase; Akt – protein kinase B; S6K1 – S6 kinase 1; TSC1/2 – tuberous sclerosis complex 1/2; mTOR (mechanistic target of rapamycin) – serine/threonine kinase; FMRP (fragile X mental retardation protein) – RNA binding protein; PP2A – protein phosphatase 2A; H-RAS – GTPase; RAF1, MEK1/2, ERK1/2 and MNK1/2 – kinases; eIF4E – factor of translation initiation; 4EBP2 – 4E-binding protein; CYFIP1 – cytoplasmic FMRP interacting protein 1. The proteinaceous products of genes whose mutations are associated with neurological disorders are shown in red. Green arrows indicate pathways of local translation activation via PP2A phosphatase and the RAS/ERK signaling pathway. The red arrow indicates blocking via the mTOR signaling pathway.

Numerous examples have been reported that impaired local translation control at a synapse brings forth various neuropsychic disorders, including ASDs, epilepsy, Parkinson’s disease, etc. (Gkogkas, Sonenberg, 2013; Buffington et al., 2014; Klein et al., 2016; Martin, 2016; Trifonova et al., 2017). Figure 2 illustrates the main regulatory events mediating the activation of local protein synthesis in dendritic spines of glutamatergic synapses of hippocampal pyramidal cells in response to the stimulation of metabotropic glutamate receptors (mGluR) on the postsynaptic membrane of excitatory synapses by glutamate. The local translation activity is controlled by the mTOR and RAS/ERK pathways (Huber et al., 2000; Darnell, Klann, 2013; Beggs et al., 2015; Chen, Joseph, 2015). 

The key element in the regulation of local cap-dependent translation at a synapse is the RNA-binding fragile X mental retardation protein, or FMRP (Feng et al., 1997). It is the target of S6 kinase and PP2A phosphatase, which are activated in response to the stimulation of mGluR receptors (Narayanan et al., 2007, 2008). When phosphorylated, it arrests translation by binding to mRNA, ribosomes, and the eIF4E translation factor (Brown et al., 1998; Napoli et al., 2008; Chen et al., 2014). Dephosphorylation disrupts FMRP linkage to its targets, resulting in, on the one hand, to accelerated mRNA translation and, on the other hand, rapid degradation of FMRP itself (Nalavadi et al., 2012). FMRP controls translation efficiency through RNA binding sites (Chen, Joseph, 2015). It directly binds to the coding and 3′-UTR mRNA sequences (Brown et al., 1998; Darnell et al., 2011) and to the L5 protein of 80S ribosomes (Chen et al., 2014). In this way, it controls transcription elongation and termination. Translation can also be repressed in 3′-UTR by physical interaction of FMRP with the 43-kDa TAR DNA-binding protein (TDP-43) (Majumder et al., 2016).

FMRP is also involved in translation regulation at its initiation step by interaction with the cytoplasmic FMRP-interacting protein 1 (CYFIP1) (Napoli et al., 2008). The current notions of mechanisms regulating translation by means of FMRP (Napoli et al., 2008; Majumder et al., 2016) presume the interaction of a single molecule of the protein with 3′-UTR via TDP-43 and with translation initiation factor eIF4E via CYFIP1. Thus, FMRP and CYFIP1 are the key regulators of translation regulation at an activated synapse. 

FMRP targets are mRNAs for proteinaceous components of the mTOR signaling pathway (PI3K kinase, PTEN phosphatase, tuberous sclerosis complex 2 (TSC2 and mTOR), PP2A phosphatase, receptor proteins (mGluR, NMDAR, and AMPAR), proteins forming the postsynaptic membrane (NLGN, SHANK, and PSD95), the ubiquitin-dependent protein degradation system (E3 ubiquitin ligase), and its own mRNA (FMR1) (Brown et al., 1998; Muddashetty et al., 2007; Gross et al., 2010; Sharma et al., 2010; Darnell et al., 2011; Ascano et al., 2012). Apparently, FMRP plays the key role in dynamic proteome regulation at an activated synapse (Zukin et al., 2009; Iacoangeli, Tiedge, 2013). 

It is known that mutations in genes encoding most of these proteins result in synapse malfunction and various disorders. Mutations in the gene for the SHANK3 protein of the postsynaptic membrane cause Phelan–McDermid syndrome; in the gene for PTEN phosphatase, Cowden’s disease; for NF1, type 1 neurofibromatosis; in the genes for GTPase, H-RAS, RAF1, and MEK1 kinase, Costello and Noonan syndromes; TSC2-TSC1, tuberous sclerosis; FMRP, fragile X syndrome; UBE3A ubiquitin-protein ligase, Angelman syndrome; and in genes for neuroligins NLGN3/4 and neurexin NRXN1, typical autism (Trifonova et al., 2016). Mutations in the Shank3 gene and its abnormal expression are also considered to cause autism, schizophrenia, and epilepsy (Peça et al., 2011; Mei et al., 2016; de Sena Cortabitarte et al., 2017; Monteiro, Feng, 2017; Fu et al., 2020). Mutations in the gene for PTEN phosphatase often bring forth various neurological diseases: macrocephaly, epilepsy, mental deficiency, and autism (Zhou, Parada, 2012; Trifonova et al., 2016).

These data suggest that synapse malfunctions are related to anomalies in local translation regulation. One of the possible synaptopathy causes is disturbed proteome stability, which hampers the formation of synapse plasticity and long-term memory (Cajigas et al., 2010). Indeed, just poor proteome stability is reported to be associated with autism and other neuropsychic disorders (Klein et al., 2016; Louros, Osterweil, 2016).

It should be mentioned that the structure-functional organization of the system regulating FMRP activity includes negative and positive feedback loops, which are instability factors in molecular systems (Mackey, Glass, 1977; Decroly, Goldbeter, 1982; Goldbeter et al., 2001; Bastos de Figueiredo et al., 2002; Likhoshvai et al., 2013, 2015, 2016, 2020; Kogai et al., 2015, 2017; Suzuki et al., 2016; Khlebodarova et al., 2017).

These regulatory loops act in different time spans. They are associated with rapid (ca. 1 min) translation activation of FMRP-dependent mRNAs via PP2A phosphatase and its rather rapid (2–5 min) arrest via the activation of S6 kinase (Narayanan et al., 2007, 2008). That is, the normal work of a synapse is supported by fine dynamic interplay among components of these signaling pathways at an activated synapse (see Fig. 2).

Analysis of dynamic features of the local translation system shows that an increase in the rate and efficiency of FMRP-dependent translation may induce instability in the local translation system, in particular, just in the physiological range of its operation (Khlebodarova et al., 2018; Likhoshvai, Khlebodarova, 2019). This result suggests that the known cases of ASDs related to the hyperactivity of the translation system at synapses (Pramparo et al., 2015; Onore et al., 2017) stem from proteome stability impairments associated with the formation of complex dynamic patterns of receptor protein synthesis in response to synapse stimulation (Khlebodarova et al., 2018, 2020). It is a brand-new insight into possible causes of synaptopathies.

It should be added that the elevated activity of mTOR signaling is a feature of not only ASDs but also other psychic and neurological diseases: Alzheimer’s disease (Pei, Hugon, 2008), epilepsy (Wong, 2010), and even Down syndrome (Troca-Marin et al., 2012). It is also presumed that elevated mTOR activity causes early senescence and age-related neurodegenerative conditions in humans (Johnson et al., 2013). 

In this regard, the hypothesis that the high copy numbers of rRNAs in some individuals are a risk factor for the development of ASDs, schizophrenia, and mental deficiency appears to be reasonable (Chestkov al., 2018; Porokhovnik, 2019; Porokhovnik, Lyapunova, 2019) on the assumption that individual variations in copy numbers of rRNA genes correlate with ribosome concentrations in a cell and the activity of the translational machinery.

## The actin cytoskeleton and neurodegenerative diseases 

**Fig. 3.**
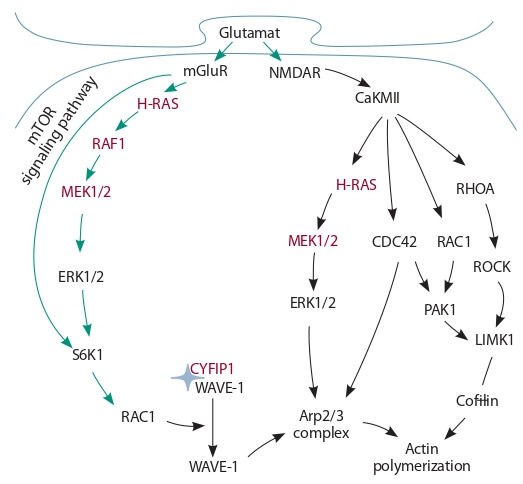
Schematic presentation of the regulation of actin cytoskeleton formation in dendritic spines of glutamatergic synapses of hippocampal pyramidal cells in response to synapse stimulation. mGluR, NMDAR – receptor proteins; H-RAS, RHOA, RAC1, CDC42 – RAS GTPases; CaKMII, MEK1/2, RAF1, ERK1/2, S6K1, PAK1, ROCK, LIMK1 – kinases; CYFIP1 – cytoplasmic FMRP interacting protein 1; WAVE-1 – WAVE-1 regulatory complex; Arp2/3 – actin binding proteins; Cofilin – actin depolymerization factor. The proteinaceous products of genes whose mutations are associated with neurological disorders are shown in red. Green arrows indicate the pathways of actin polymerization regulation via mTOR and S6K1, black – via CaKMII signaling.

The actin cytoskeleton structure determines the morphology of dendritic spines in nerve cells. Its rearrangements by rapid assembly of actin monomers (G actin) to filaments (F actin) and inverse disassembly are essential for the formation of synaptic plasticity and long-term memory (Penzes, Rafalovich, 2012; Basu, Lamprecht, 2018). Disturbances in the mechanisms regulating the formation of F actin filaments and dendritic spine structure are thought to be associated with neurodegenerative disorders: Alzheimer’s disease, schizophrenia, and autism (Bamburg, Bernstein, 2016; Borovac et al., 2018; Forrest et al., 2018; Ben Zablah et al., 2020; Lauterborn et al., 2020). Fig. 3 illustrates the major regulatory events underlying actin cytoskeleton rearrangements in dendritic spines of glutamatergic synapses in hippocampal pyramidal cells, which are activated in response to the action of glutamate on metabotropic glutamate receptors mGluR and ionotropic glutamate receptors NMDAR (N-methyl-d-aspartate receptor) on the postsynaptic membrane of excitatory synapses. 

The induction of actin filament formation and filament stabilization at an activated synapse depends substantially on the activity of cofilin and the WAVE regulatory complex, which is controlled by S6K, LIMK1, and PAK1 kinases via signaling pathways mediated by the RAS family of small 

GTPases: H-RAS, RhoA, Rac1, and Cdc42 (Tapon, Hall, 1997; Rex et al., 2009; Ip et al., 2011; Chen et al., 2017; Schaks et al., 2018). The operation of these signaling pathways at an activated synapse depends greatly on fast de novo synthesis of Rho GTPases (Briz et al., 2015). Arrest of protein synthesis in dendritic spines of hippocampal cells completely suppresses the stimulation of RhoA GTPase, cofilin phosphorylation, and actin polymerization (Briz et al., 2015). A mutation in the Fmr1 gene, which encodes the FMRP protein, the key local transcription regulator, completely suppresses the physiological stimulation of GTPase Rac1 and its effector PAK1 kinase, disrupting the stabilization of actin filaments at hippocampal cell synapses (Chen et al., 2010).

Proceeding from the above, the activity of RAS GTPases controlling the formation and stabilization of actin filaments in dendritic spines depends directly on their de novo synthesis, i. e., on the activity of mTOR and FMRP-dependent local translation. It is conjectured that unstable local translation (Khlebodarova et al., 2018, 2020; Likhoshvai, Khlebodarova, 2019), also results in hypo- or hyperactivity of RAS, which, in turn, causes aberrations in the structure of dendritic spines and neurological disorders associated therewith (Ba et al., 2013; Pyronneau et al., 2017; Zamboni et al., 2018; Nishiyama, 2019).

Rac1 GTPase plays the key role in the regulation of the heteropentameric WAVE regulatory complex. The activity of this GTPase depends much on S6K and mTOR1 kinases. Normally, the WAVE complex is inactive, but its interaction with Rac GTPase induces its dissociation into two subcomplexes: CYFIP1-containing and WAVE1-containing (Derivery et al., 2009). The latter interacts with the Arp2/3 (actin-related proteins) complex and induces actin polymerization, as in Fig. 3 (Cory, Ridley, 2002; Millard et al., 2004; Abekhoukh, Bardoni, 2014; Molinie, Gautreau, 2018). 

The disintegration of the WAVE complex and aberrant WAVE1 activation cause epileptic encephalopathy (Nakashima et al., 2018; Zhang et al., 2019; Zweier et al., 2019; Schaks et al., 2020). This may happen in cases of abnormal stoichiometric control of WAVE component synthesis (Abekhoukh et al., 2017) or mutations disrupting the interaction between WAVE1 and CYFIP2 (Nakashima et al., 2018; Zhang et al., 2019; Zweier et al., 2019; Schaks et al., 2020).

It should be noted that CYFIP1, being one of the main components of the WAVE regulatory complex, is also involved in translation regulation at its initiation step by interaction with the RNA-binding FMRP protein (Napoli et al., 2011). Thus, the mechanisms regulating local translation and actin cytoskeleton rearrangements in neural dendritic spines are additionally interlinked via the CYFIP1 protein (De Rubeis et al., 2013). 

## Conclusion

Analysis of presently available data shows the mechanisms regulating the local translation system at synapses and dynamic rearrangements of the actin cytoskeleton in dendritic spines of nerve cells, which play the central role in the formation of various types of synapse plasticity and long-term memory, are closely linked to each other and to the activity of the YAP/TAZ mechanosensor. This sensor can indirectly, via mTOR and S6K kinase, affect both translation efficiency and the state of actin filaments in dendritic spines (Tapon, Hall, 1997; Tumaneng et al., 2012; McCarthy, 2013; Reddy et al., 2013; Briz et al., 2015; Hu et al., 2017; Seo, Kim, 2018). 

It is well substantiated that mTOR hyperactivity and functional aberrations in practically every component of the local translation system and of the machinery controlling rearrangements of the actin cytoskeleton in dendritic spines can cause numerous neurodevelopment disorders of various origins (Pei, Hugon, 2008; Wong, 2010; Johnson et al., 2013; Pramparo et al., 2015; Onore et al., 2017; Pyronneau et al., 2017; Trifonova et al., 2017; Nakashima et al., 2018; Nishiyama, 2019; Zhang et al., 2019).

Theoretical analysis of the dynamic features of local translation system operation presented in (Khlebodarova et al., 2018, 2020; Likhoshvai, Khlebodarova, 2019) suggests that one of the possible mechanisms of neurological disorders arising under chronic mechanical stress is the abnormal hyperactivity of mTOR and local translation at the synapse. It induces the dynamic instability of de novo protein synthesis at the activated synapse. 

Thus, it is obvious that chronic mechanical stress may be one of the risk factors for synaptopathies and neurodegenerative diseases because of mTOR hyperactivation, which disturbs proteome stability, much needed for proper synapse plasticity and long-term memory (Klein et al., 2016; Louros, Osterweil, 2016). 

## Conflict of interest

The authors declare no conflict of interest.
